# Integrative analysis from multi‐centre studies identifies a function‐derived personalized multi‐gene signature of outcome in colorectal cancer

**DOI:** 10.1111/jcmm.14403

**Published:** 2019-05-29

**Authors:** Jie Sun, Hengqiang Zhao, Shuting Lin, Siqi Bao, Yan Zhang, Jianzhong Su, Meng Zhou

**Affiliations:** ^1^ School of Ophthalmology & Optometry and Eye Hospital, School of Biomedical Engineering Wenzhou Medical University Wenzhou P. R. China

**Keywords:** colorectal cancer, integrative analysis, personalized gene signature

## Abstract

Colorectal cancer (CRC) is highly heterogeneous leading to variable prognosis and treatment responses. Therefore, it is necessary to explore novel personalized and reproducible prognostic signatures to aid clinical decision‐making. The present study combined large‐scale gene expression profiles and clinical data of 1828 patients with CRC from multi‐centre studies and identified a personalized gene prognostic signature consisting of 46 unique genes (called function‐derived personalized gene signature [FunPGS]) from an integrated statistics and function‐derived perspective. In the meta‐training and multiple independent validation cohorts, the FunPGS effectively discriminated patients with CRC with significantly different prognosis at the individual level and remained as an independent factor upon adjusting for clinical covariates in multivariate analysis. Furthermore, the FunPGS demonstrated superior performance for risk stratification with respect to other recently reported signatures and clinical factors. The complementary value of the molecular signature and clinical factors was further explored, and it was observed that the composite signature called IMCPS greatly improved the predictive performance of survival estimation relative to molecular signatures or clinical factors alone. With further prospective validation in clinical trials, the FunPGS may become a promising and powerful personalized prognostic tool for stratifying patients with CRC in order to achieve an optimal systemic therapy.

## 1 INTRODUCTION

Colorectal cancer (CRC) is the third most common type of cancer diagnosed in men and women, and is the leading cause of morbidity and mortality worldwide. The estimated number of new cases and mortalities because of CRC in 2018 in USA are 140 250 and 50 630, respectively.^1^ Surgery combined with radiotherapy, chemotherapy and/or targeted therapies are the most common treatments for CRC, which are mainly based on the American Joint Committee on Cancer (AJCC) tumor‐node‐metastasis (TNM) staging system. However, the TNM staging system is not sufficient for treatment decisions and prognosis prediction of patients with CRC.^2^, ^3^, ^4^ For example, patients with stage IIB CRC tend to show poor prognosis, with a 5‐year relative survival rate of 46%‐61% compared with those with stage IIIA (~70%).^5^, ^6^ This limitation of TNM staging indicates an increasing and urgent need for identifying novel biomarkers to improve the outcome and treatment of patients with CRC.

With the application of muti‐omics technologies to study CRC, it was demonstrated that CRC is of high heterogeneity at the intertumoral and intratumoral levels. Patients with CRC often have variable prognosis and treatment responses, even in tumours that are histologically identical.^7^ Advances in molecular profiling have enabled a better understanding of CRC development and provide additional clinically relevant prognostic information beyond the current classic staging system.^8^ During the past years, considerable substantial efforts have been made to identify gene expression‐based biomarkers for predicting prognosis in patients with CRC. Although these efforts were an important step to guide clinical decision‐making, few recently proposed signatures have been incorporated into clinical practice. A previous systematic review performed comparison analysis and revealed limited overlap across different signatures and poor prognostic performance across different independent datasets.^9^ The potential issues preventing the translation of in silico data into clinical practice include (a) the fact that these existing signatures were usually generated from a small sample size or a single dataset; (b) did not account for biological heterogeneity and technical biases across different datasets, which led to overfitting and concentrating on the discovery dataset; and (c) insufficient independent validation. These limitations highlighted the requirement for adequate sample size and multi‐institutional patient cohorts for sufficient statistical power when trying to identify a robust prognostic signature.^10^ Another major concern for low reproducibility is that these signatures only focus on statistical values and often fail to incorporate the biological rationale, thus leading to the incorporation of unrelated genes, which are correlative rather than causative.^11^, ^12^ Therefore, it is necessary to explore novel personalized and reproducible prognostic signatures based on multi‐institutional cohorts of patients with CRC of sufficient size to aid clinical decision‐making and improve the outcomes of patients with CRC.

The present study combined large‐scale gene expression profiles and clinical data of patients with CRC from multi‐centre studies and developed a novel computational framework to identify a function‐derived personalized gene signature (FunPGS) for improving outcome. This prognostic signature was validated in multiple independent datasets across different technology platforms, and its performance was assessed in comparison to recently proposed signatures.

## 2 MATERIALS AND METHODS

### 2.1 Patient cohorts and study design

To obtain datasets of patients with CRC, a comprehensive database search for CRC studies was conducted, which included patient datasets according to following selection criteria: (a) Datasets with genome‐wide transcriptional profiles and clinicopathological annotations; and (b) datasets with large sample size (n > 50). Finally, a total of 1828 patients from 10 public CRC datasets were analysed in the present study, including nine microarray datasets from the Gene Expression Omnibus (GEO, https://www.ncbi.nlm.nih.gov/geo/) database and one RNA‐Seq dataset of The Cancer Genome Atlas (TCGA) from the UCSC Xena project (https://xena.ucsc.edu/).

To minimize undesired bias across the datasets, seven datasets profiled on the same array platform (Affymetrix U133 Plus 2.0 Array) were selected as the meta‐training cohort. The remaining three patient datasets profiled with different platforms and outcome measure were utilized as an independent testing cohort for validating the prognostic value of the signature. Detailed information about these 10 CRC datasets is shown in Table 1 and Table S1.

**Table 1:** CRC patient datasets enrolled in the study

Datasets	Use	Platform	Number of patients	Outcome
GSE39582	Meta‐training cohort	HG‐U133_Plus_2	579	OS
GSE17536	Meta‐training cohort	HG‐U133_Plus_2	177	OS
GSE72970	Meta‐training cohort	HG‐U133_Plus_2	124	OS
GSE38832	Meta‐training cohort	HG‐U133_Plus_2	122	DSS
GSE39084	Meta‐training cohort	HG‐U133_Plus_2	70	OS
GSE29621	Meta‐training cohort	HG‐U133_Plus_2	65	OS
GSE17537	Meta‐training cohort	HG‐U133_Plus_2	55	OS
TCGA	Independent testing cohort	Illumina HiSeq 2000	321	OS
GSE14333	Independent testing cohort	HG‐U133_Plus_2	226	DFS
GSE33113	Independent testing cohort	HG‐U133_Plus_2	89	RFS

Abbreviations: DFS, Disease‐free survival; DSS, Disease‐specific survival; OS, Overall survival; RFS, Recurrence‐free survival.

### 2.2 Pre‐processing of profiling data

Raw microarray datasets (.CEL files) from the GEO database were pre‐processed and normalized using the Robust Multi‐array Average (RMA) algorithm for background correction, log_2_‐transformation and quantile normalization using the R package ‘affy’. All microarray probes were mapped to Entrez Gene ID, and the mean value of multiple probes mapping to the same gene ID was used to represent the gene expression level using the R package ‘limma’. To avoid systematic measurement bias, each microarray datum was normalized independently by Z‐score transformation. The log_2_‐transformed RSEM‐normalized count data of level 3 released gene expression data derived from the Illumina HiSeq platform were extracted as gene expression measurements from the UCSC Xena project (https://xena.ucsc.edu/).

### 2.3 Gene set function analysis

Gene Ontology (GO) function enrichment analysis of the prognostic gene sets was performed using the R package ‘clusterProfiler’.^13^ GO terms with *P* < 0.01 were considered to be significantly different and were selected for further analysis. Significantly enriched GO terms were clustered and visualized using the Enrichment Map plugin in Cytoscape.^14^ Functional similarity among different sets of enriched GO terms was computed using the R package ‘GOSemSim’.^15^

### 2.4 Development of a function‐derived personalized gene signature

**Figure 1:**
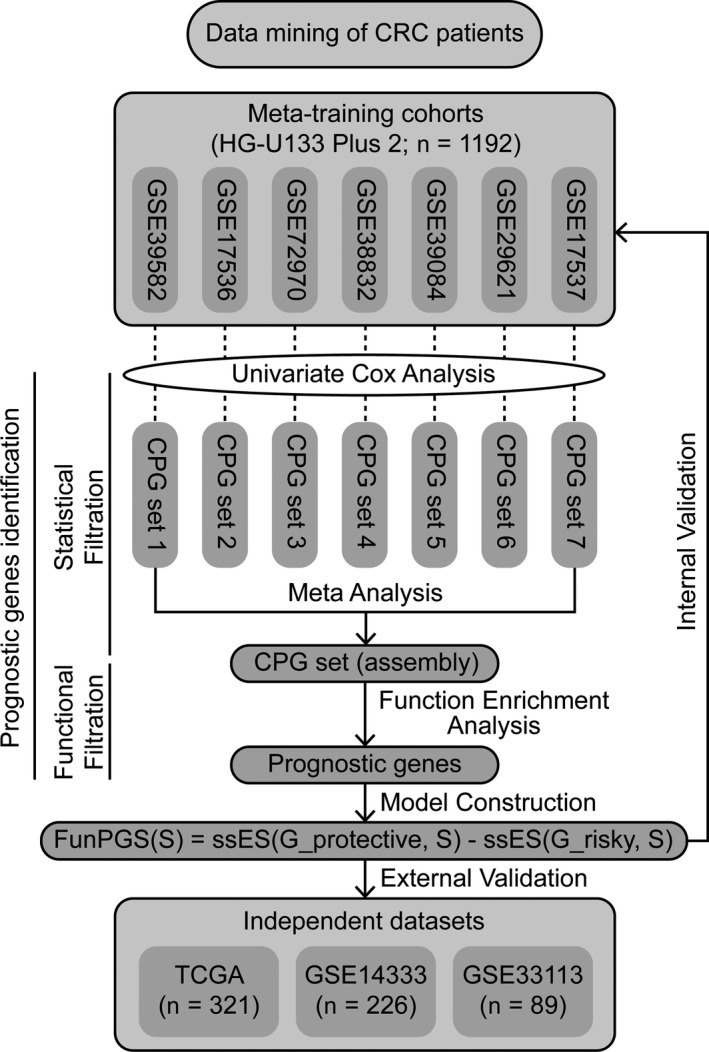
Schematic representation of the computational workflow to derive and validate a function‐derived gene signature as a personalized prognostic predictor of outcome in patients with colorectal cancer

The workflow describing the development of the FunPGS is illustrated in Figure 1. The FunPGS was developed based on an improved version of the single‐sample gene set enrichment analysis (ssGSEA) scoring method,^16^, ^17^ as follows:

$$ssES\left(G,S\right)=\sum_{i=1}^{N}\left[P_{\mathrm{hit}}\left(G,S,i,w\right)-P_{\mathrm{miss}}\left(G,S,i\right)\right]$$

$$P_{\mathrm{hit}}\left(G,S,i,w\right)=\sum_{g_{j}\in G,j\leq i}\frac{{\left|r\left(g_{j}\right)\right|}^{w}}{\sum_{g_{j\in G}}{\left|r\left(g_{j}\right)\right|}^{w}}$$

$$P_{\mathrm{miss}}\left(G,S,i\right)=\sum_{g_{j}\in G,j\leq i}\frac{1}{\left(N-N_{G}\right)}$$

$$FunPGS\left(S\right)=ssES\left(G_{\mathrm{protective}},S\right)-ssES\left(G_{\mathrm{risky}},S\right)$$

In the above equations, G represents the prognostic gene set, $G_{\mathrm{protective}}$ represents the protective prognostic gene set, $G_{\mathrm{risky}}$ represents the risk‐associated prognostic gene set, S represents the single patient sample, $r\left(g_{j}\right)$ represents the rank of gene *j* in the prognostic gene set and $N_{G}$ is the number of prognostic genes. Patients with a high FunPGS displayed better outcomes than those with a low FunPGS.

### 2.5 Statistical analysis

Survival analysis, including univariate and multivariate analyses with Cox proportional hazards regression and Kaplan‐Meier analysis with log‐rank test was performed using the R package ‘survival’. Hazard ratios (HRs) and 95% confidence intervals (CIs) were calculated. Harrell's concordance index (C‐index) was calculated for each dataset to evaluate its prognostic performance using the R package ‘survcomp’. Time‐dependent ROC curves and AUC at 3‐ and 5‐years were calculated to assess the predictive performance of molecular signature in comparison with clinical prognostic factors (stage and age) using the R package ‘timeROC’. Meta‐analysis was performed using the R package ‘meta’. Because of potential heterogeneity among clinical samples and studies, heterogeneity among studies was assessed using the Higgins’ *I* ^2^ and Q statistics. When heterogeneity existed among studies (*P* < 0.1 and *I* ^2^ > 50%), random‐effect models were used for the meta‐analyses. Otherwise, the fixed‐effect model was employed. Similarity of gene membership between each prognostic signature pair was assessed by the Jaccard index. All statistical analyses were performed using R (v3.3.3) and Bioconductor.

## 3 RESULTS

### 3.1 Consistency evaluation of prognostic gene sets from different patient datasets

**Figure 2:**
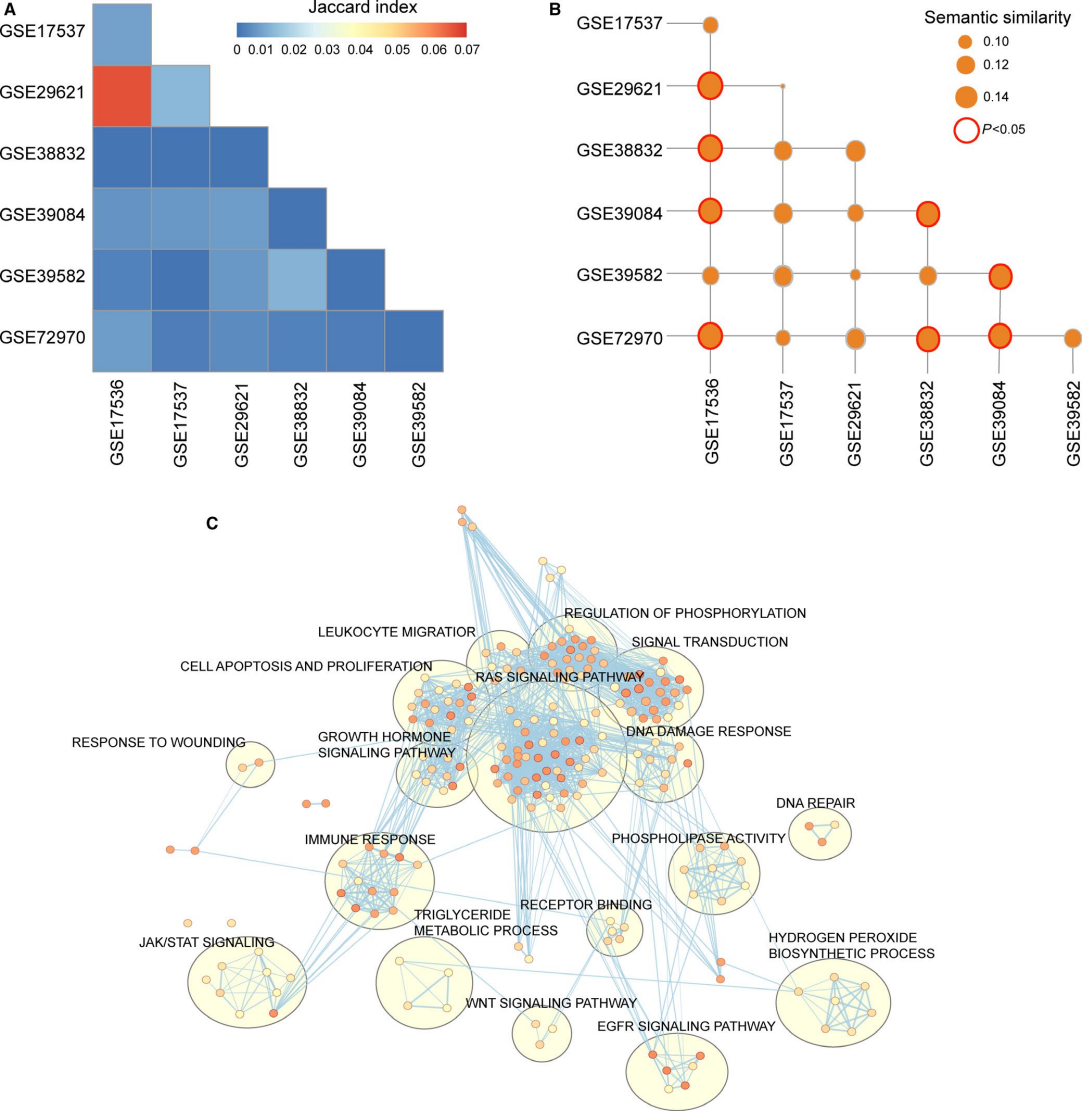
Consistency evaluation of prognostic gene sets from different patient datasets. (A) Heatmaps of Jaccard indices comparing the similarity across different prognostic gene sets. (B) Heatmaps of GO semantic similarity across different prognostic gene sets. (C) Functional enrichment map of GO terms of 46 prognostic genes. Node size represents the number of genes in the GO terms. Colour intensity is proportional to enrichment significance. GO, Gene Ontology

The association of genes with survival was first assessed in each dataset of the meta‐training cohort using univariate Cox regression analysis followed by multivariate analysis adjusted by clinical variables including stage, gender and age. This resulted in seven prognostic gene sets encompassing 1391 genes (383 from GSE17536, 326 from GSE17537, 134 from GSE29621, 64 from GSE38832, 161 from GSE39084, 36 from GSE39582 and 347 from GSE72970) (Table S2). The Jaccard index was calculated to determine the degree of overlap between the prognostic gene sets, and a number of intersections were observed (Figure 2A), indicating the low reproducibility of prognostic genes from a single patient dataset, as shown previously.^9^, ^18^ Then, GO enrichment analysis was performed for each prognostic gene set, and the functional similarity among enriched GO terms was calculated for each prognostic gene set pair. Compared with random signatures, these prognostic gene sets derived from a single patient dataset exhibited significantly high functional consistency (Figure 2B).

### 3.2 Construction and definition of the FunPGS

**Figure 3:**
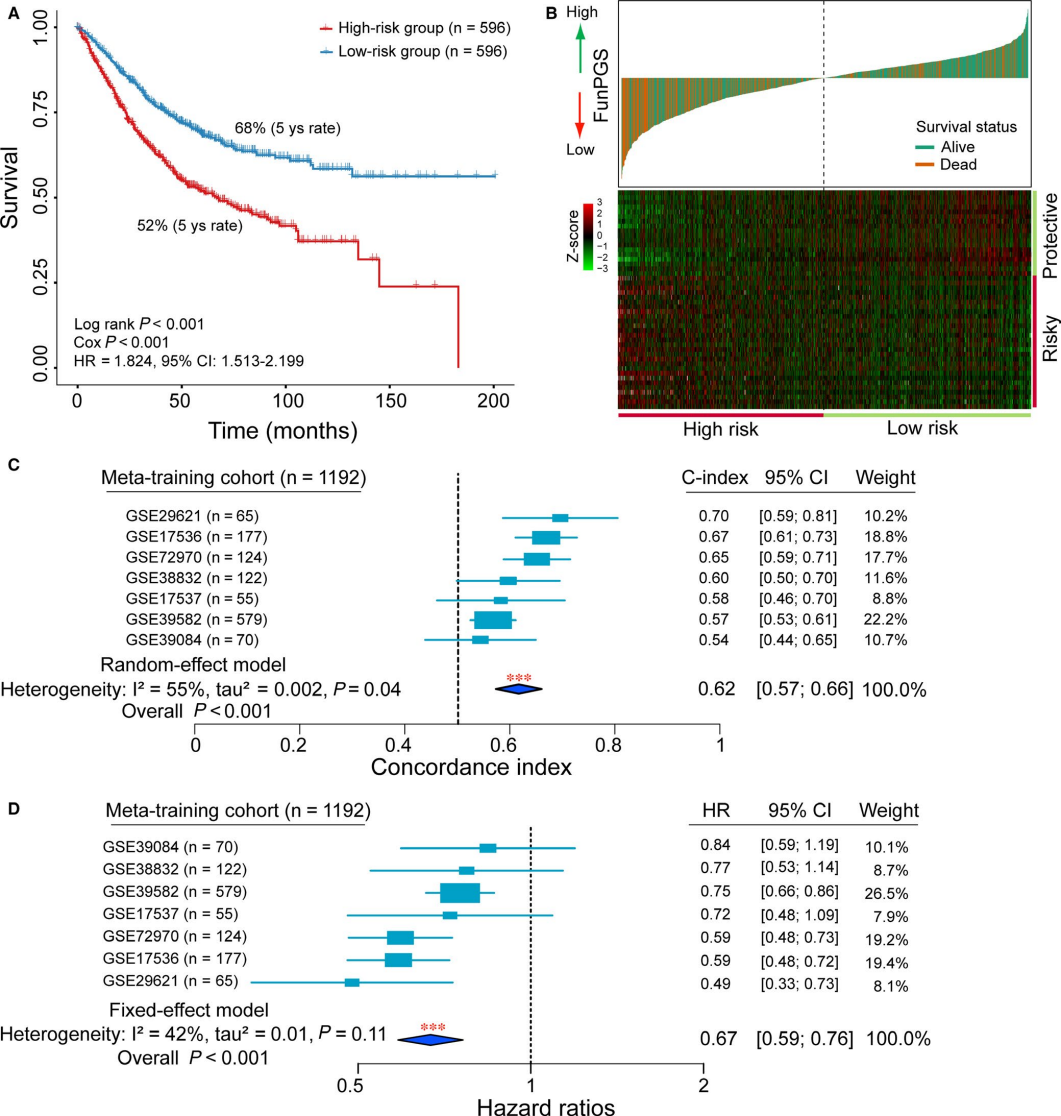
Assessment of prognostic performance of the FunPGS in the meta‐training cohort. (A) Kaplan‐Meier curves of patients with colorectal cancer at low or high risk stratified by the FunPGS in the meta‐training cohort. (B) Distribution of the risk score and expression heatmaps of 46 genes in the prognostic signature. (C) Forest plot visualizing the C‐index of the FunPGS in all the seven datasets of the meta‐training cohort. The horizontal line corresponds to the 95% CI and the vertical line indicates a C‐index of 0.5. The blue diamond shows the random‐effects meta‐analysis summary of CIs over the seven datasets (C‐index = 0.62, 95% CI = 0.57‐0.66, *P* < 0.001). (D) Forest plot visualizing HRs of univariate analysis of the FunPGS in all the seven datasets of the meta‐training cohort. The horizontal line corresponds to the 95% CI and the pink vertical line indicates a HR of 1.0. The blue diamond shows the fix‐effects meta‐analysis summary of HRs over the seven datasets (HR = 0.67, 95% CI = 0.59‐0.76, *P* < 0.001). C‐index, Harrell's concordance index; CI, confidence interval, HR, hazard ratio

Based on the above observations, the present study developed a computational, statistical function‐derived workflow to construct a robust personalized gene signature (Figure 1), which resulted in 46 prognostic genes from a biological perspective in the meta‐training cohort, including 18 protective prognostic genes (*CEBPA*, *KLHDC3*, *FITM2*, *GALM*, *CYP1A1*, *ANKS4B*, *IL12A*, *CC2D1A*, *ZC4H2*, *PRLR*, *VANGL2*, *NDRG2*, *CCL22*, *GORASP1*, *ST6GAL1*, *TRAF1*, *L3MBTL4* and *ARHGEF11*) and 28 risk‐associated prognostic genes (*CDKN2A*, *NDRG1*, *FAM3C*, *CHD2*, *FZD10*, *DSG3*, *ACSL4*, FOXD1, FLT1, KLK5, WSB1, MYOF, KRT6C, GRB10, ANXA2, HOXC6, ITGA3, *KRT6A*, *KRT6B*, *MARK3*, *MSH4*, *GULP1*, *KLK7*, *KLK6*, *ANXA8*, *TJP1*, *PTTG1IP* and *DLG5*). These 46 prognostic genes were significantly enriched in 203 GO terms, which could be grouped into several functional clusters, including RAS signalling pathway, growth hormone signalling pathway, JAK/STAT signalling pathway, WNT signalling pathway, EGFR signalling pathway, immune response, DNA repair, DNA damage response, cell apoptosis and proliferation (Figure 2C). Then, the FunPGS was constructed from 1192 patients in the meta‐training cohort as described in Figure 1. The FunPGS divided these 1192 patients from the meta‐training cohort into two risk groups by the median score, namely patients with low FunPGS (high risk) and patients with high FunPGS (low risk). As shown in Figure 3A, patients predicted as ‘high risk’ experienced significantly shorter survival compared with those predicted as ‘low risk’ (high risk vs. low risk, 5‐year survival 52% vs. 68%, log‐rank test, *P* < 0.001; HR = 1.824, 95% CI = 1.513‐2.199, *P* < 0.001). Patients with low FunPGS exhibited a higher risk of succumbing to disease than those with high FunPGS (number of mortalities 274 vs. 186, χ^2^ test, *P* < 0.001) (Figure 3B). With increasing FunPGS, patients expressed higher levels of protective prognostic genes and lower levels of risk‐associated prognostic genes (Figure 3B).

The C‐index for the FunPGS was estimated in each dataset of the meta‐training cohort separately and was integrated using meta‐analysis for evaluation of the overall prognostic value of the FunPGS. The forest plot using the random‐effect model indicated a significantly favourable prognostic value for the FunPGS, and the relevant meta‐analysis revealed a C‐index of 0.62 (95% CI = 0.57‐0.66, *P* < 0.001) (Figure 3C). A univariate analysis was conducted for each dataset of the meta‐training cohort separately, and a meta‐analysis was performed on the meta‐training cohort. As shown in the forest plot of Figure 3D, the FunPGS was significantly associated with survival time, with a fixed‐effect pooled HR of 0.67 (95% CI = 0.59‐0.76, *P* < 0.001), which confirmed the strong prognostic value of the FunPGS on outcome.

### 3.3 External validation of the FunPGS on three independent datasets across different technology platforms

**Figure 4:**
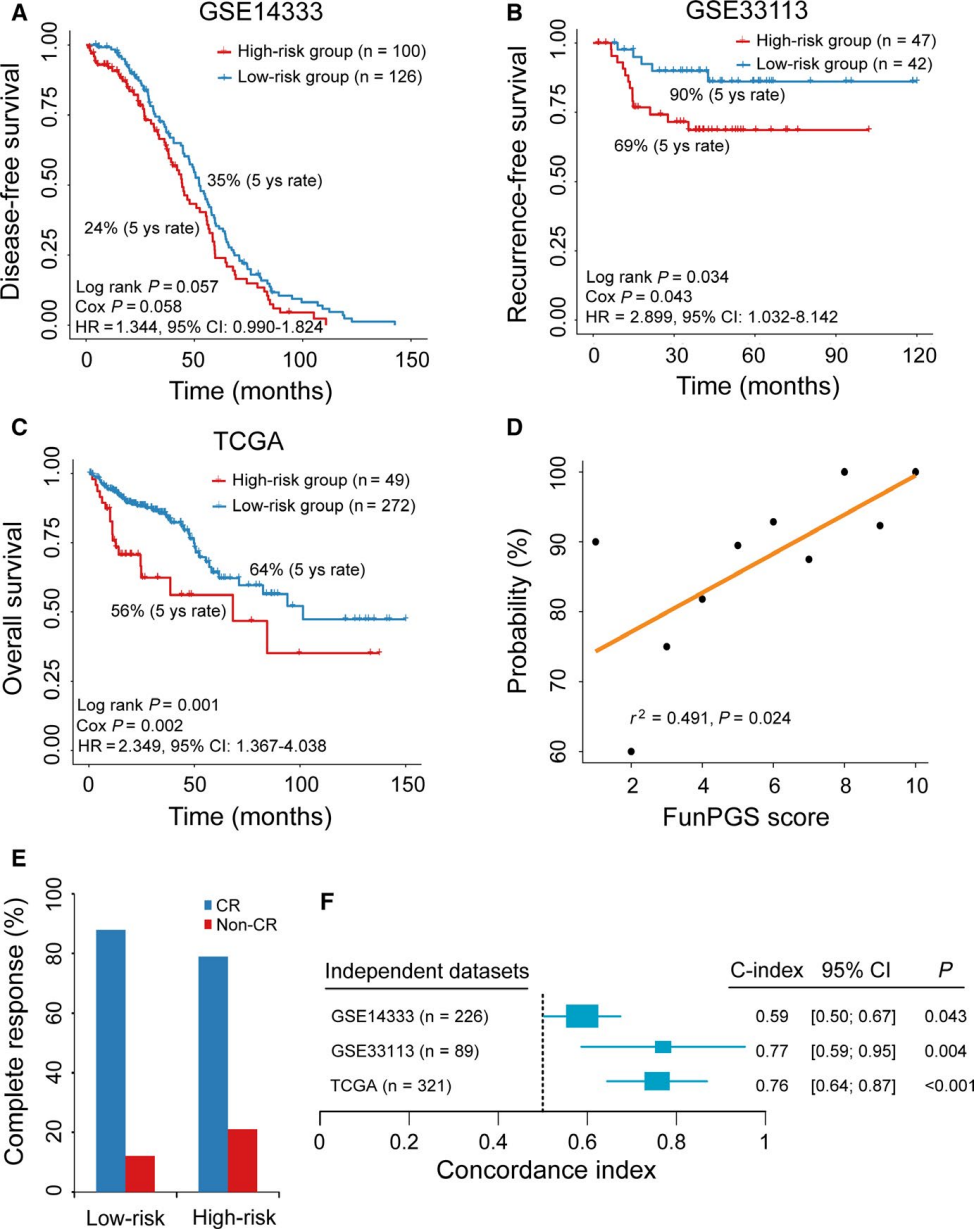
Prognostic independent evaluation of the FunPGS. Kaplan‐Meier curves of patients with colorectal cancer at low or high risk stratified by the FunPGS in the (A) GSE14333, (B) GSE33113 and (C) TCGA datasets. (D) Correlation of the FunPGS with CR. The Pearson's correlation coefficient was calculated to assess the association between the FunPGS and the likelihood of CR The straight line depicts the least squares linear regression line fitted to the data points. (E) Differences in CR ratios between the high and low‐risk groups stratified by the FunPGS. (F) Forest plot visualizing the C‐index of the FunPGS in all independent validation datasets. The horizontal line corresponds to the 95% confidence interval and the pink vertical line indicates a C‐index of 0.5. CR, complete response; C‐index, Harrell's concordance index

The ability of the FunPGS for stratifying patients with CRC and different prognosis was first confirmed in two validation datasets with microarray platforms. For the GSE14333 dataset, disease‐free survival (DFS) was used as the survival endpoint. The FunPGS significantly stratified patients into high‐ and low‐risk groups in terms of DFS. Patients in the high‐risk group had significantly shorter DFS than those in the low‐risk group (median DFS, 44.4 vs. 52.2 months, log‐rank test, *P* = 0.057) (Figure 4A). The 5‐year DFS rate of patients in the high‐ and low‐risk groups was 24 and 35%, respectively. When applying the FunPGS to the GSE33113 dataset with recurrence‐free survival (RFS) as the survival endpoint, there was a significant difference in RFS between the two groups, with a 5‐year RFS of 90% and 69% in patients with high FunPGS and low FunPGS, respectively (log‐rank test, *P* = 0.034) (Figure 4B). In univariate analysis, the HRs of high vs. low risk for DFS and RFS were 1.344 (*P* = 0.058, 95% CI = 0.990‐1.824) in the GSE14333 dataset and 2.899 (*P* = 0.043, 95% CI = 1.032‐8.142) in the GSE33113 dataset.

Further validation of the FunPGS was conducted on an independent RNA‐seq dataset of 321 patients with CRC from TCGA. According to the FunPGS, these 321 patients were divided into a high‐risk group (n = 49) and a low‐risk group (n = 272). Consistently with the findings described above, high FunPGS was significantly associated with improved outcome (HR = 2.349, 95% CI = 1.367‐4.038, *P* = 0.002). The median overall survival (OS) of the high‐ and low‐risk groups was 68.2 and 101.4 months, respectively (log‐rank test, *P* = 0.001) (Figure 4C). The present study further examined whether the FunPGS was correlated with the probability of complete response (CR) by plotting the percentage of patients achieving CR against the FunPGS score. As shown in Figure 4D, there was a significantly positive correlation between the FunPGS and CR (Pearson's correlation coefficient *r* ^2^ = 0.491, *P* = 0.024) (Figure 4D). Out of 143 patients with CRC and known response information, 124 (86.7%) patients achieved CR, with 109 patients (87.9%) being in the low‐risk group and 15 patients (12.1%) in the high‐risk group (Figure 4E).

The C‐index for the FunPGS was calculated in each of the three independent datasets. As shown in Figure 4F, the FunPGS yielded a significant prognostic value, exhibiting a high C‐index in all the independent datasets (GSE14333, C‐index = 0.59, *P* = 0.043; GSE33113, C‐index = 0.77, *P* = 0.004; and TCGA, C‐index = 0.76, *P* < 0.001) (Figure 4F).

### 3.4 Independence of the FunPGS from other clinicopathological factors

**Figure 5:**
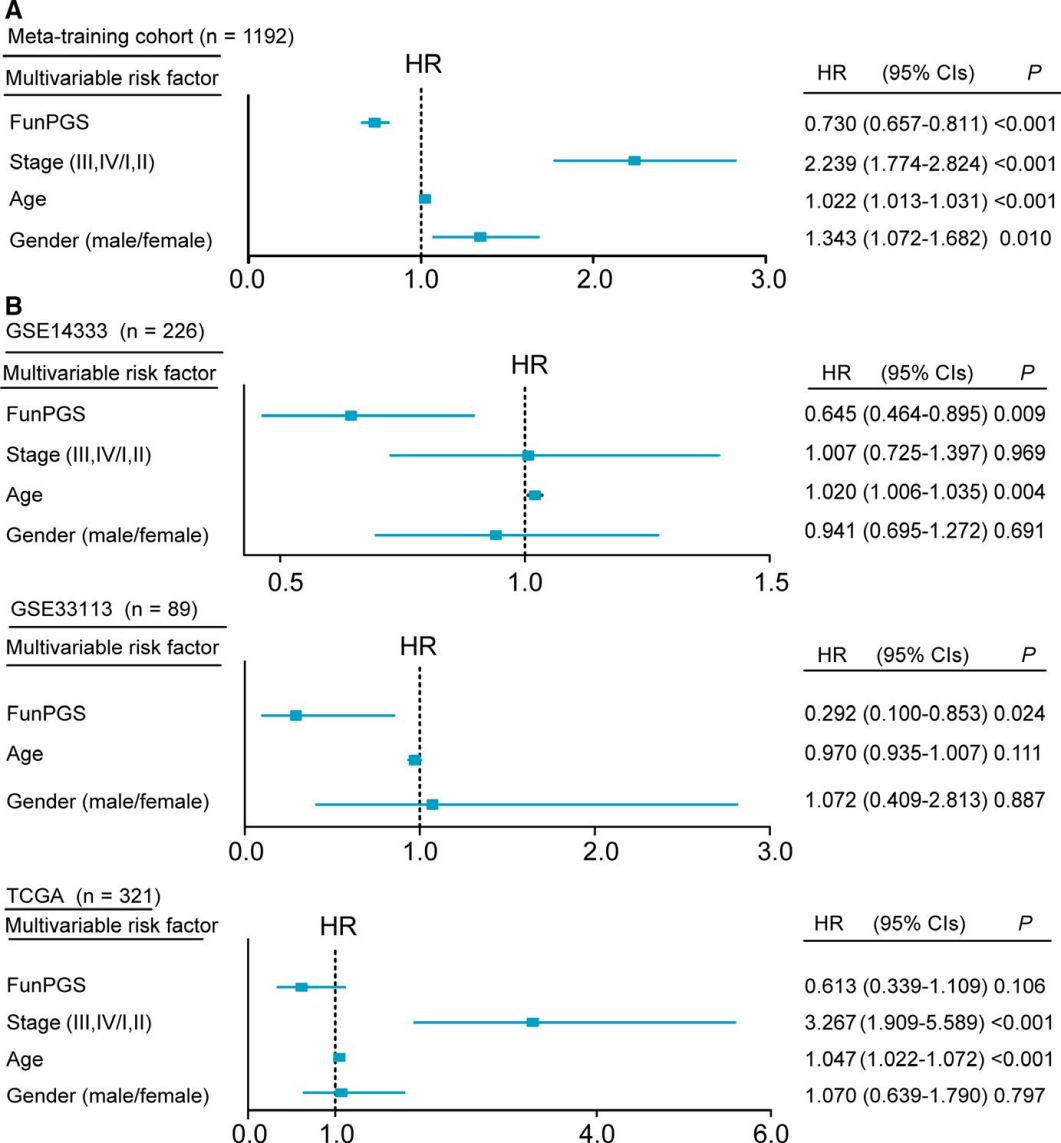
Independence of the FunPGS from other clinicopathological factors. Multivariate analysis of clinicopathological factors and the FunPGS was performed. (A) Forest plot visualizing the HRs of multivariate analysis in the meta‐training cohort and all the independent validation datasets (B). The horizontal line corresponds to the 95% CI and the pink vertical line indicates a HR of 1.0. CI, confidence interval, HR, hazard ratio

To evaluate whether the FunPGS was independent from other clinical or pathological factors, multivariate Cox regression analysis was conducted in the meta‐training cohort and three independent datasets using the following factors as categorical variables: FunPGS score (high vs. low), tumor stage (III‐IV vs. I‐II), age (≥60 vs. <60 years old) and gender (male patients vs. female patients). In the meta‐training cohort, the HR of high FunPGS vs. low FunPGS for OS was 0.73 (*P* < 0.001, 95% CI = 0.657‐0.811) upon adjusting for other clinicopathological factors (Figure 5A), indicating that the FunPGS still maintained a significantly independent correlation with OS.

Repeating the multivariate analysis for the three independent datasets revealed that high FunPGS was consistently associated with improved outcome, while low FunPGS was associated with poor outcome in all three independent datasets (GSE14333, HR = 0.645, 95% CI = 0.464‐0.895, *P* = 0.009; GSE33113, HR = 0.292, 95% CI = 0.100‐0.853, *P* = 0.024; and TCGA, HR = 0.613, 95% CI = 0.339‐0.1.109, *P* = 0.106), whereas all other clinicopathological factors failed to show a consistent association with outcome (Figure 5). These results from multivariate analysis indicated that the predictive power of the FunPGS is independent of other clinicopathological factors and, furthermore, it outperformed other clinicopathological factors.

### 3.5 Prognostic performance of the FunPGS in comparison with five previously published gene signatures

**Figure 6:**
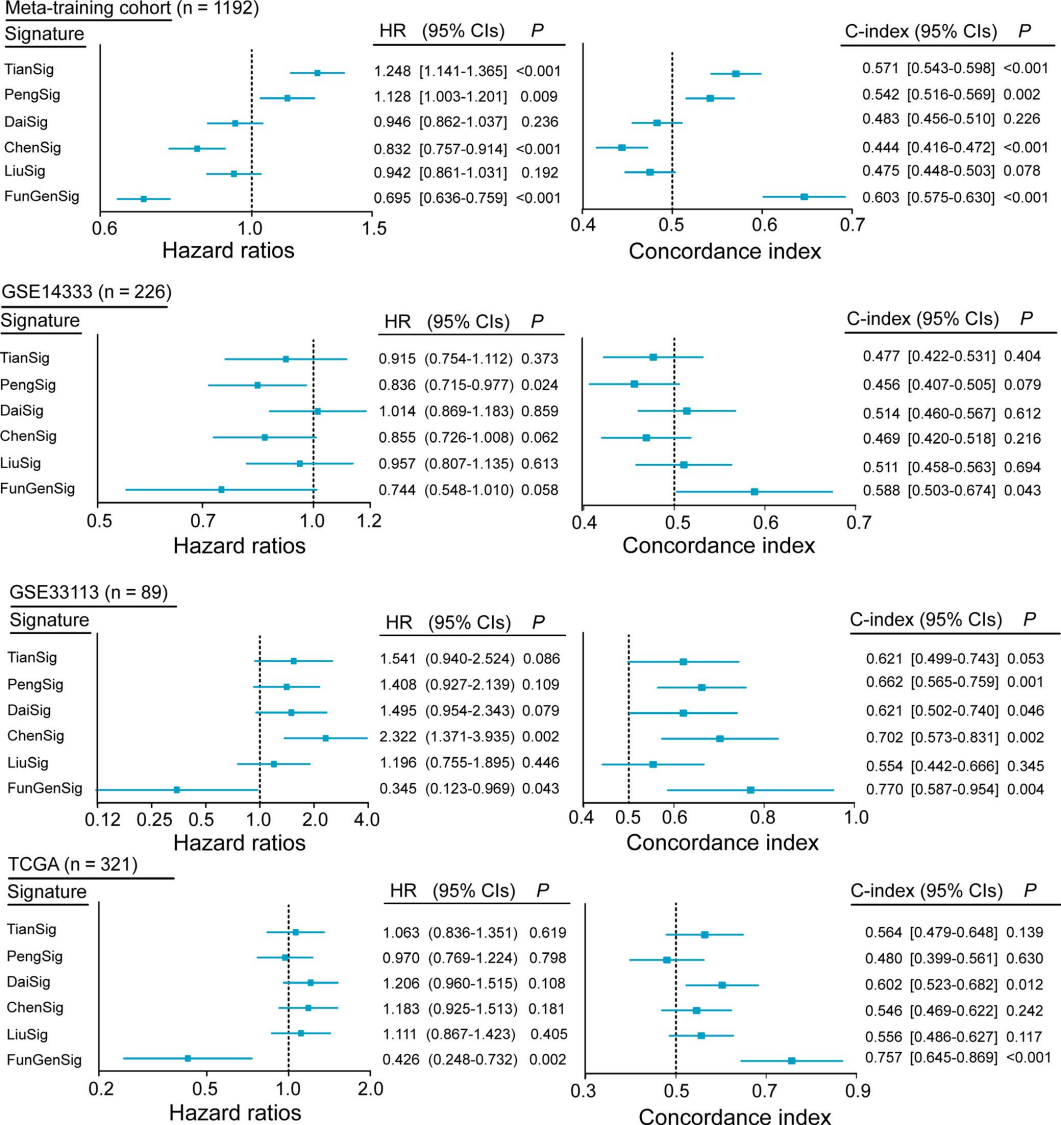
Prognostic comparison of the FunPGS with recently published signatures. Performance of the FunPGS in comparison with five previously published gene signatures. Forest plot visualizing the HRs of univariate analysis and C‐index of the FunPGS in the meta‐training cohort and all the independent validation datasets. The horizontal line corresponds to the 95% confidence interval and the pink vertical line indicates a HR of 1.0 and a C‐index of 0.5. HR, hazard ratio; C‐index, Harrell's concordance index

In the present study, five gene expression‐based signatures associated with outcome of patients with CRC were collected retrospectively from previous studies, namely TianSig,^19^ PengSig,^20^ DaiSig,^21^ ChenSig 22 and LiuSig.^23^ The present study assessed and compared the prognostic power of the FunPGS and the other five previously published gene signatures for each dataset by estimating the HR from univariate analysis and C‐index. The results from univariate analysis suggested that only the FunPGS retained a significant and consistent association with improved outcome (meta‐training cohort, HR = 0.695, 95% CI = 0.636‐0.759, *P* < 0.001; GSE14333, HR = 0.744, 95% CI = 0.548‐1.010, *P* = 0.058; GSE33113, HR = 0.345, 95% CI = 0.123‐0.969, *P* = 0.043; and TCGA, HR = 0.426, 95% CI = 0.248‐0.732, *P* = 0.002), while the other gene signatures failed to have a consistent association with outcome (Figure 6). The comparative analysis was repeated with C‐index as the performance criterion and similar conclusions were obtained. As shown in the forest plots of C‐index, the FunPGS exhibited significantly higher C‐index than the other gene signatures in each dataset (Figure 6). These results suggested that the FunPGS had a better predictive performance than the five previously published gene signatures.

### 3.6 Integrated prognostic signature obtained by combining the FunPGS with clinical factors

**Figure 7:**
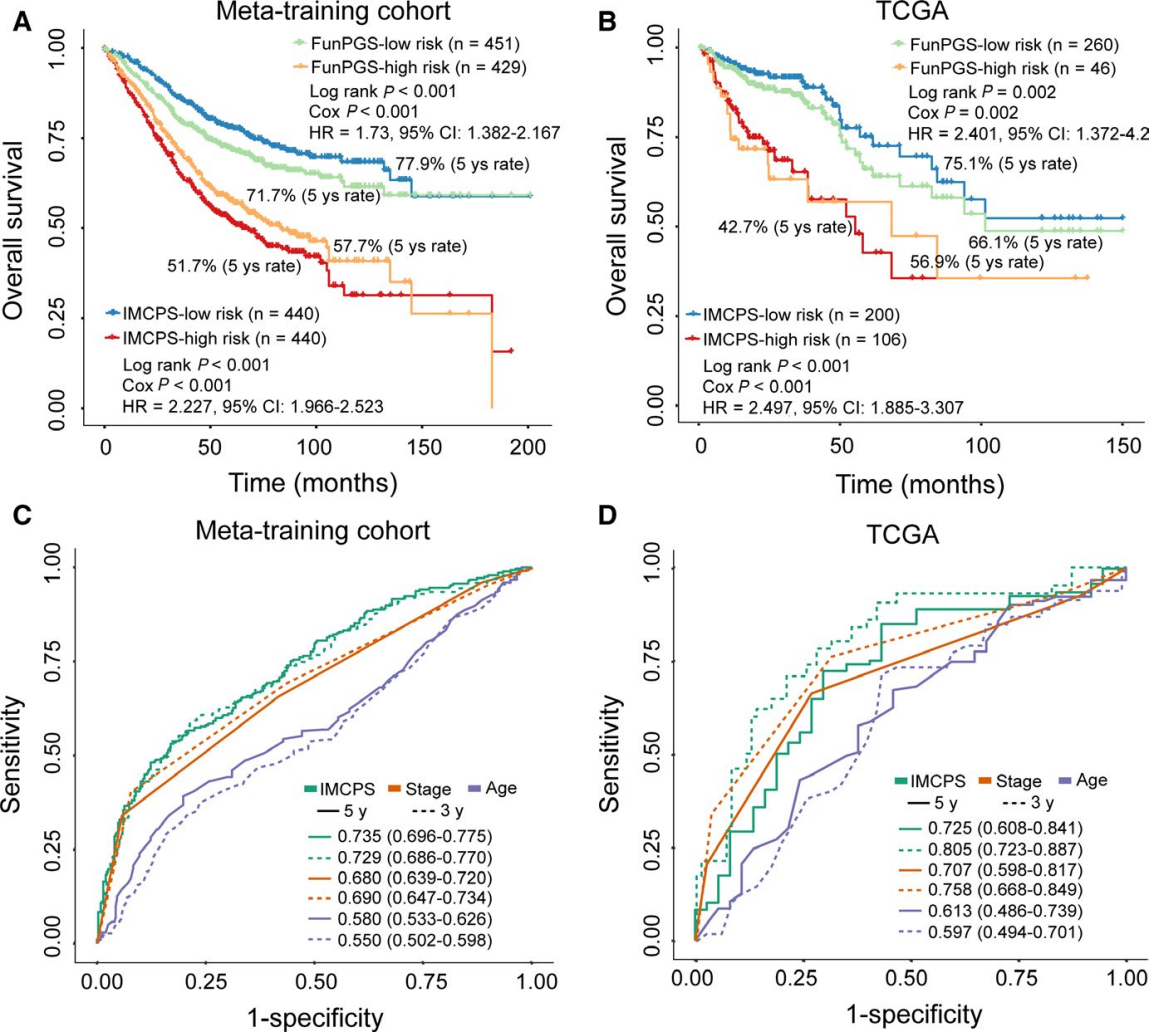
Assessment of the prognostic performance of the IMCPS. Kaplan‐Meier curves of overall survival for patients with colorectal cancer at low or high risk stratified by the FunPGS or the IMCPS in the (A) meta‐training cohort and (B) TCGA dataset. Time‐dependent ROC curves at 3 or 5 years for the FunPGS or IMCPS in the (C) meta‐training cohort and (D) TCGA dataset

The AJCC TNM staging system and age are well‐known important prognostic factors.^24^, ^25^ In the multivariate analysis conducted in the present study, TNM stage and age remained significantly associated with survival besides the FunPGS in the meta‐discovery and TCGA cohorts, indicating the independent and complementary value of TNM stage and age. Therefore, the FunPGS, TNM stage and age were combined to construct an integrated molecular and clinical factors‐based prognostic signature (IMCPS), and its prognostic value was tested. The IMCPS was quantified by subjecting the FunPGS, TNM stage and age to a multivariate Cox regression model in the meta‐discovery cohort as follows: (−0.0001 × FunPGS) + (0.7792 × TNM stage) + (0.0236 × age). The median score of the IMCPS derived from the meta‐discovery cohort was used as a cut‐off value to stratify patients into low‐ or high‐risk groups. As shown in Figure 7, the IMCPS had higher separation than the FunPGS alone between the high and low‐risk groups in the meta‐training cohort and the independent TCGA dataset (Figure 7A and 7). The present study also compared the IMCPS with TNM stage and age by time‐dependent ROC analysis and observed that the AUC of the IMCPS was significantly higher than TNM stage and age at 3‐year and 5‐year OS in both the meta‐training cohort and the independent TCGA dataset. Thus, the IMCPS greatly improved the predictive performance of survival estimation (Figure 7C and 7).

## 4 DISCUSSION

Current treatment guidelines and prognosis prediction of patients with CRC in clinical practice rely principally on histological characteristics and tumour stage. However, these factors have shown marked limitations towards personalized medicine in CRC.^12^ Patients with CRC that display similar histopathological features exhibited significantly different prognosis or variable response to therapy. This may be partly because of heterogeneous prognosis and treatment responses, which may arise from molecular heterogeneity.^8^, ^26^ Therefore, reliable and independent molecular biomarkers capable of differentiating patients with CRC into different prognostic groups are required. Based on the availability of large‐scale and multi‐centre molecular profiles and clinical data, the present study first conducted a systematic evaluation of prognostic genes based only on statistical considerations. Our results indicated that prognostic genes identified from a single dataset are hard to recur in other datasets but achieved more functional consistency (Figure 2). However, previous studies of CRC gene expression signatures failed to incorporate biological significance and only focused on statistical power during biomarker selection, thus leading to low reproducibility. Therefore, we developed a novel computational framework to identify prognostic genes based on a combination of statistical and functional reasons, which increased reliability and overcame the limits of sample size. Starting with an integrative analysis of multiple datasets using a statistics and function‐derived approach, 46 robust prognostic genes were identified, which were involved in multiple key carcinogenic biological processes and pathways, suggesting that these prognostic genes correlate with prognosis and may be causative in tumorigenesis.

Despite previous attempts to develop gene signatures for outcome prediction, the majority of reported prognostic signatures were constructed based on a linear score method using weighted expression levels of prognostic genes. Thus, these previously reported gene signatures demonstrated insufficient power to personalized outcome prediction, whereas the expression level of an individual patient must be normalized together with different samples in advance, which is impractical in a clinical setting.^27^ To meet patient‐level needs and accelerate clinical practice, the present study integrated these 46 robust prognostic genes into a personalized gene signature (FunPGS) based on the improved ssGSEA method. The FunPGS was able to characterize prognostic risks only involved in pairwise comparison of enrichment score based on the expression levels of protective and risk‐associated prognostic genes within a sample, thus enabling a personalized outcome prediction for patients with CRC at the individual level. Furthermore, the FunPGS represents differential activity levels of key biological processes and pathways rather than simply different expression levels of individual genes.^16^, ^17^

The FunPGS was constructed in a meta‐training cohort comprising 1192 patients from seven datasets, which overcomes the weakness of small sample size and a single simple source. Limitations in the application of previously proposed signatures have demonstrated the importance of rigorous validation and reproducibility for medical applications.^28^ The FunPGS effectively discriminated patients with CRC and significantly different survival and was successfully validated in a completely independent TGCA RNA‐seq dataset, suggesting that the FunPGS is robust and insensitive to technical biases that are inherent to measurements by different platforms. It is well known that the outcome of patients with CRC has improved by the combined treatment of surgical resection and adjuvant chemotherapy. However, because of molecular heterogeneity, a group of patients with a relatively low incidence of recurrence may be over‐treated with unnecessary adjuvant chemotherapy. By contrast, numerous patients still face disease relapse or distant metastases despite therapy because of the heterogeneous chemotherapeutic response.^8^ With further validation, the FunPGS also demonstrated greater ability to predict RFS and DFS in two independent datasets in terms of risk of relapse or distant metastases, indicating that the FunPGS has a great potential to facilitate the selection of patients with CRC who have a high risk of relapse or distant metastases and who may benefit from additional systemic therapy.

Currently, the clinical prognostic of patients with CRC relies largely on the AJCC TNM staging system, although the number of prognostic biomarkers is increasing. Recent studies have demonstrated that, besides TNM stage, age and gender are important prognostic factors for survival of patients with CRC.^24^, ^25^ Therefore, it is important to assess whether the prognostic performance of the FunPGS is independent of these known clinical factors or associated with it. By applying multivariate analysis in the meta‐training and independent validation datasets, the FunPGS not only demonstrated risk‐stratification ability independent of these known clinical factors, but also is superior to the performance of these known clinical factors for outcome predictions. These results also suggested that the FunPGS can add clinically relevant prognostic and predictive information beyond these known clinical prognostic factors.

Comparing the FunPGS with previously reported gene signatures, no significant overlap among them was observed. Such variability among these signatures may be because of the use of different platforms, samples sizes and methodology. Therefore, the present study attempted to evaluate the prognostic value of the FunPGS by comparing it with five recently reported signatures. It was observed that all five previously published signatures confirmed their prognostic value only in limited datasets. Instead, prognostic value of the FunPGS was not only consistently validated in all the datasets, but also outperformed these five previous signatures. This result may further emphasize the importance of the use of biological rationale during biomarker selection and signatures construction.^11^

In conclusion, the present study developed a novel computational framework based on a combination of statistical and functional rationale, and identified a personalized gene signature that is predictive of OS and other measures of clinical outcome for patients with CRC in multiple datasets across different centres and platforms. However, it should be noted that, despite the fact that the FunPGS achieved better performance than available clinical factors and previous signatures, further investigation in additional available datasets and prospective clinical trials are required to ensure its reliability and actual use in individualized management of patients with CRC.

## CONFLICT OF INTEREST

The authors declare that they have no competing interests.
